# Visible Light Induced Exciton Dynamics and *trans*-to-*cis* Isomerization in Azobenzene Aggregates: Insights from Surface Hopping/Semiempirical Configuration Interaction Molecular Dynamics Simulations

**DOI:** 10.1021/acsomega.3c09900

**Published:** 2024-02-09

**Authors:** Evgenii Titov

**Affiliations:** Institute of Chemistry, Theoretical Chemistry, University of Potsdam, Karl-Liebknecht-Straße 24-25, 14476 Potsdam, Germany

## Abstract

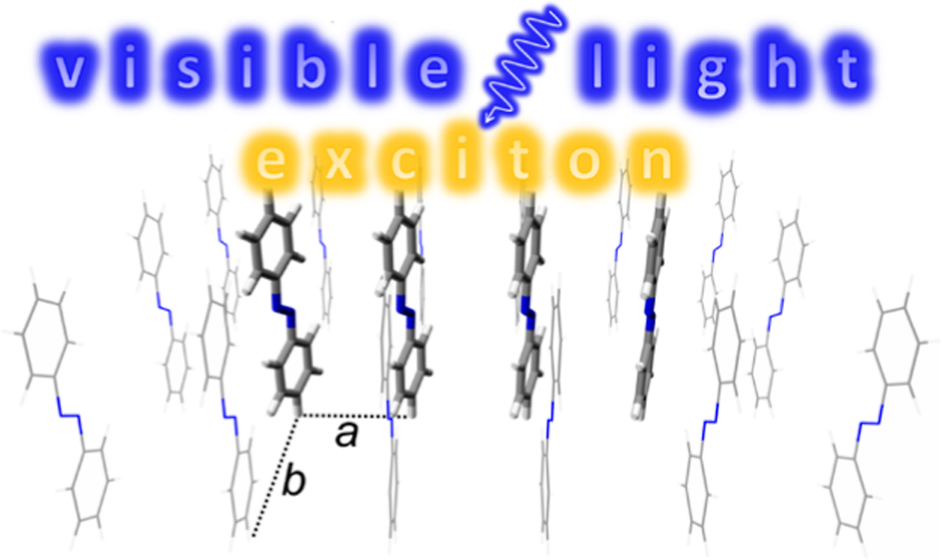

Assemblies of photochromic molecules feature exciton states, which govern photochemical and photophysical processes in multichromophoric systems. Understanding the photoinduced dynamics of the assemblies requires nonadiabatic treatment involving multiple exciton states and numerous nuclear degrees of freedom, thus posing a challenge for simulations. In this work, we address this challenge for aggregates of azobenzene, a prototypical molecular switch, performing on-the-fly surface hopping calculations combined with semiempirical configuration interaction electronic structure and augmented with transition density matrix analysis to characterize exciton evolution. Specifically, we consider excitation of azobenzene tetramers in the nπ* absorption band located in the visible (blue) part of the electromagnetic spectrum, thus extending our recent work on dynamics after ππ* excitation corresponding to the ultraviolet region [Titov, *J. Phys. Chem. C***2023**, *127*, 13678–13688]. We find that the nπ* excitons, which are initially strongly localized by ground-state conformational disorder, undergo further (very strong) localization during short-time photodynamics. This excited-state localization process is extremely ultrafast, occurring within the first 10 fs of photodynamics. We observe virtually no exciton transfer of the localized excitons in the nπ* manifold. However, the transfer may occur via secondary pathways involving ππ* states or the ground state. Moreover, we find that the nπ* quantum yields of the *trans*-to-*cis* isomerization are reduced in the aggregated state.

## 1 Introduction

Chromophores assembled together, such as in molecular crystals or nanoscale assemblies, give rise to formation of molecular excitons—electronically excited states of molecular aggregates.^1−3^ The molecular excitons are key players in operation of organic optoelectronic devices^4^ as well as in the natural process of photosynthesis.^5^ A particularly interesting situation arises if the monomer itself—the building block of a molecular aggregate—is a photochromic system. In this case, aggregation may affect not only photophysics of the chromophore (*e.g.*, electronic spectra^6^) but also its photochemistry (*e.g.*, hinder isomerization^7,8^).

A prototypical example of the photochromic system is azobenzene, undergoing *trans* ↔ *cis* isomerizations upon illumination with light in ultraviolet (UV) and visible regions of the electromagnetic spectrum.^9^ Arrangement of numerous azobenzene units in proximity to each other takes place in molecular crystals,^6^ liquid crystals,^10^ self-assembled monolayers (SAMs),^11,12^ micelles of azobenzene-containing surfactants,^13,14^ surfactant–polymer complexes,^15,16^ and other supramolecular systems and architectures.^17,18^ Distinct spectroscopic signatures (such as spectral shifts and changes in absorbance) originating from azobenzene aggregation have been observed experimentally, *e.g.*, for SAMs^19^ and micellar solutions.^14^ Moreover, several studies have reported first-principles calculations of exciton states of azobenzene aggregates and SAMs.^20−25^ However, these studies address exciton states for fixed nuclear configurations of the aggregates. To go beyond this single-geometry picture, it is necessary to account for ground-state conformational (geometrical) disorder (induced by thermal fluctuations) and excited-state dynamical effects.

The effect of the conformational disorder in the electronic ground state may be modeled either by performing ground-state molecular dynamics (GSMD) simulations (usually applying a thermostat to mimic temperature of environment) or by sampling Wigner function corresponding to the vibrational ground state of the electronic ground state.^26^ The vertical absorption spectra and exciton localization are then computed for a set of selected geometries. In the case of large (multichromophoric) molecules or molecular aggregates, the GSMD approach is usually used because the Wigner function is computed in practice in the harmonic approximation, and hence, the Wigner sampling may be problematic for anharmonic modes, which are present in the complex systems. Recently, we have investigated the effect of the conformational disorder on the exciton states of a model azobenzene tetramer (with *trans*-azobenzene units assembled in an H-aggregate fashion) by time-dependent density functional theory (TD-DFT) calculations performed at nuclear configurations generated by the GSMD DFT approach.^27^ We have found that the ground-state conformational disorder leads to partial localization of the ππ* excitons (with a localization degree being dependent on the temperature) and strong localization (to a single monomer) of the nπ* excitons.^27^ Here, we should recall that the absorption spectrum of *trans*-azobenzene includes a weak lower energy nπ* band and an intense higher energy ππ* band.^28,29^

Further, illumination of molecular assemblies with light excites them from the electronic ground state to the electronically excited state(s), inducing subsequent exciton dynamics. These dynamics require, in general, nonadiabatic treatment, *i.e.*, coupled electron-nuclear dynamics involving many potential energy surfaces should be described. Over the past few years, the exciton dynamics in multichromophoric systems have been modeled using various approaches to nonadiabatic dynamics, including surface hopping (SH),^30−35^ multiconfiguration time-dependent Hartree (MCTDH)^36,37^ and multilayer MCTDH,^38−40^ Ehrenfest and multiconfigurational Ehrenfest (MCE),^41,42^ and symmetrical quasi-classical windowing model applied to the classical Meyer–Miller vibronic Hamiltonian (SQC/MM).^43,44^ However, only a few studies have been devoted to the exciton dynamics in azobenzene aggregates. Sangiogo Gil *et al.* used SH combined with an exciton model to simulate Frenkel exciton dynamics in an azobenzene dimer (azobenzenophane)^45^ and an azobiphenyl monolayer.^46^ Recently, we have employed a supermolecule approach to investigate exciton dynamics in several azobenzene tetramers (both free and embedded in a SAM-like environment) after ππ* excitation.^47^ The exciton evolution was explored using a transition density matrix (TDM) analysis, allowing one to judge on the spatial localization of excitons during SH dynamics.^30,31,33,34,48−52^

In this work, we study the exciton dynamics after nπ* excitation, *i.e.*, induced with visible light excitation, for the models introduced in our previous work on the photodynamics after ππ* excitation.^47^ While the ππ* absorption band of azobenzene is more intense than the nπ* band, the latter is located in the visible region of the electromagnetic spectrum, which is of preference for applications requiring lower energy photons, *e.g.*, for use in biological systems.^53^ Indeed, one of the central goals of contemporary research in the field of photoswitches is to enable efficient isomerization with low-energy photons.^54−56^ Here, using nonadiabatic dynamics simulations, we study how the corresponding low-energy excitons formed in azobenzene aggregates evolve in time.

The paper is organized as follows. In the next section (Section 2), we describe the used models and methods. In Section 3, we present and discuss the results. Specifically, in Section 3.1, we describe the absorption spectrum of the aggregates and initial exciton localization. Next, in Section 3.2, we discuss exciton dynamics initiated by nπ* excitation. After that, excited-state lifetimes and quantum yields of the *trans* → *cis* isomerization are presented in Section 3.3. Section 4 concludes the work.

## 2 Models and Methods

**Figure 1:**
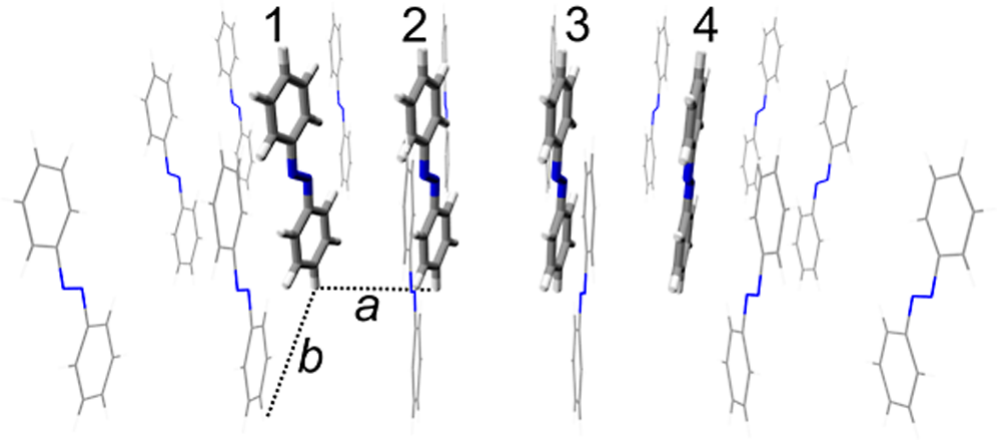
Model of SAM 5.5 Å. Shown is the starting geometry for the Langevin ground-state trajectory. Thick molecules are the QM part, and the thin molecules are the MM part. The numbering of the QM molecules is shown on top. The parameters of the rectangular lattice are *a* = 5.50 Å and *b* = 9.43 Å. Further models considered in this work are SAM 3.5 Å (*a* = 3.50 Å and *b* = 6.00 Å) and “free” tetramers with *a* = 5.50 Å and *a* = 3.50 Å, which are not surrounded by the thin MM molecules (formally *b* = ∞). Perspective projection is used in this figure.

We considered the models used in our recent work.^47^ Namely, these models are “free” tetramers of stacked azobenzenes differing by the nearest-neighbor distance (3.5 and 5.5 Å) and “constrained” tetramers embedded in a perimeter of further azobenzene molecules, similarly to the situation realized in SAMs (these latter models are called SAMs in what follows). The SAM 5.5 Å model is shown in Figure 1. All other studied models may be obtained from this model by changing lattice parameters and, in the case of the free tetramers, by removing the perimeter molecules. The lattice parameters are *a* = 5.50 Å and *b* = 9.43 Å for SAM 5.5 Å and *a* = 3.50 Å and *b* = 6.00 Å for SAM 3.5 Å (the *a*/*b* ratio is the same in both cases, *a*/*b* ≈ 0.583). The SAM models are described using a quantum mechanics/molecular mechanics (QM/MM) approach: The central tetramer (thick molecules in Figure 1) is the QM part, and the perimeter (thin) molecules form the MM part. Further visualizations of the studied systems are provided in ref (47) (see Figures 1 and S1 there). We also note that these models represent an extension of our earlier models based on a QM dimer.^57,58^

To model nonadiabatic dynamics induced by visible light (nπ*) excitation, we use the approach applied in ref (47) to study relaxation after UV (ππ*) excitation. In this approach, the electronic structure of the QM tetramer is modeled with configuration interaction singles (CIS) based on molecular orbitals (MO) obtained from a self-consistent field calculation with floating occupation (FO) numbers^59^ using the Austin model 1 (AM1)^60^ that was reparameterized (r) for azobenzene.^61^ The method is abbreviated as rAM1/FOMO–CIS in what follows. For the CIS calculations, a restricted active space of eight highest occupied molecular orbitals (HOMO) and four lowest unoccupied molecular orbitals (LUMO) was used. This active space includes orbitals originating from HOMO–1 (π), HOMO (n), and LUMO (π*) of a monomer (the orbitals are shown in Figure S2 of ref (47)). In total, 65 Slater determinants are used to construct electronic wave functions.

In addition, to better describe noncovalent interactions, van der Waals (vdW) interaction terms, described with the Lennard-Jones potential, are added between atoms of different monomers (3456 pairwise potentials in total, for the QM part).^62^ The atomic vdW parameters were taken from the OPLS-AA force field.^63^ The parameters for atom pairs were calculated by taking a geometric mean of the atomic parameters. The used atomic parameters are σ_C_ = 3.55 Å, σ_H_ = 2.42 Å, σ_N_ = 3.25 Å, ε_C_ = 0.07 kcal/mol, ε_H_ = 0.03 kcal/mol, and ε_N_ = 0.17 kcal/mol. The MM part of the SAM models interacts with the QM part by the same vdW interaction. We note that we do not include the QM–MM electrostatic interaction (between partial charges). Moreover, the MM molecules were kept fixed during MD simulations. In addition, for the QM part (for all models), the C and H atoms in the para-position on one end (two atoms per monomer) were kept fixed, imitating mounting to a surface.

To sample initial conditions for SH simulations, ground-state Langevin MD trajectories at *T* = 300 K from ref (47) were used. For each system, the geometries and velocities were selected from these 20 ps long trajectories every 100 fs, starting at 5 ps, which results in 151 initial conditions per system. In comparison to ref (47), we (almost) doubled the number of trajectories to improve statistics when averaging over a swarm of trajectories (76 initial conditions were used before^47^). We should note here that computation time per trajectory could be reduced by considering fewer electronic states because nπ* states are located lower in energy than the ππ* states (see below).

The vertical absorption spectra were then calculated for the selected geometries using rAM1/FOMO–CIS, and the obtained stick spectra were broadened as
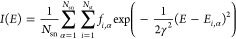
1Here, *I* is the intensity, *E* is the excitation energy, *N*_sn_ = 151 is the number of selected snapshots, *N*_st_ = 20 is the number of excited singlet states, and *E*_*i*,α_ and *f*_*i*,α_ are the excitation energy and oscillator strength, respectively, for the S_0_ → S_*i*_ transition, for snapshot α, and γ = 0.18598 eV (1500 cm^–1^) is a broadening parameter (the same value as used in ref (47)). The brightest state among the nπ* states (S_1_–S_4_) was selected as the initial state for SH calculations. We should recall here that the monomeric S_0_ → S_1_ transition is dark for the ground-state minimum geometry, but it acquires nonzero (albeit small) oscillator strength upon geometric distortions resulting from ground-state MD simulations in our case.^64^ In the tetramer, the nπ* monomeric state (S_1_) is split into four states, S_1_–S_4_. Comparing the oscillator strengths of the transitions to these exciton states from the ground state of the tetramer (for a given initial geometry), we select the state with the highest oscillator strength as an initial state for a SH run.

The nonadiabatic dynamics were modeled using the trajectory SH approach^65^ combined with the semiempirical configuration interaction method,^66^ namely, rAM1/FOMO–CIS introduced above. The SH trajectories were propagated for 10 ps with a time step of 0.1 fs. The energy-based decoherence correction^67^ was used to remedy overcoherence of the original SH algorithm [with constant *C* = 0.1 hartree; see eq 17 in ref (67)]. The time-dependent electronic wave function was propagated using the local diabatization scheme.^48,66^ The nuclei were propagated classically on the on-the-fly calculated adiabatic rAM1/FOMO–CIS PESs. The hopping probabilities were calculated using the prescription by Aguilera-Porta *et al.* described in the appendix of ref (68). We note that the local diabatization scheme is suited to propagate the electronic wave function in the presence of trivial crossings occurring for multichromophoric systems with excitonically weakly coupled states.^69,70^ However, there is no unique way to calculate hopping probabilities in the framework of local diabatization,^70,71^ and the accuracy of population dynamics may depend on the way the hopping probabilities are calculated.^71^ Nine of the lowest singlet states (S_0_–S_8_) were included in the SH simulations. Apart from the ground state (S_0_) and the nπ* states (S_1_–S_4_), we also include the ππ* states S_5_–S_8_ originating from the monomeric S_2_ state to allow for upward nπ* → ππ* nonadiabatic transitions and to check if the ππ* states play a role in photodynamics induced with visible light (nπ* excitation).

In all MD simulations, we used the so-called added potential (added to each of the four molecules of the tetramer), which corrects the low AM1 N-inversion barriers and phenyl rotations about the N–C bonds.^61^ The added potential is applied to all considered potential energy surfaces and is used throughout the SH calculations. The state-specific corrections developed in ref (61) were not used because they were derived specifically for monomeric adiabatic states.

Adiabatic electronic state populations were computed as fractions of trajectories in the state of interest. The quantum yield Φ was computed as the ratio of the number of the reactive trajectories (*i.e.*, those undergoing *trans* → *cis* isomerization) to the number *N*_t_ of trajectories that reached the ground state within 10 ps. The standard error was calculated as the standard deviation of the sample proportion, . The reactive trajectories were identified by monitoring the change in the CNNC dihedral angles from ∼180 to ∼0°.

The exciton dynamics were traced using the TDM analysis presented in our previous work.^47^ Below, we recount this analysis for completeness. The reduced one-particle spinless TDM is defined as^72^
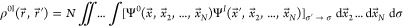
2Here, Ψ^0^ is the ground-state electronic wave function and Ψ^*I*^ is the excited current (active) state electronic wave function, *x⃗* collects spatial  and spin σ variables of an electron, and *N* is the number of electrons. We note that the electronic wave functions depend parametrically on the nuclear coordinates **R**(*t*) (which, in turn, depend on time in quantum-classical trajectory-based methods), but we do not write explicitly this dependence for the sake of brevity. We also assume the wave functions to be real.

The electronic adiabatic wave functions are linear combinations of the unexcited and singly excited Slater determinants Φ_*K*_
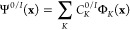
3Here, **x** collects the variables of all electrons.

Substituting eq 3 into eq 2, one can rewrite the TDM as an expansion in MO products, and further expressing MOs φ_*i*_ as linear combinations of atomic orbitals (AOs) η_μ_, , as an expansion in AO products
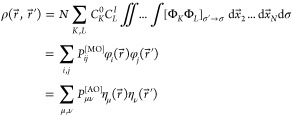
4Here, **P**^[MO]^ and **P**^[AO]^ are TDMs in the MO and AO basis, respectively. In our case, the **P**^[MO]^ matrix has a size of 12 × 12 (because there are 8 occupied and 4 virtual orbitals in the active space), and the **P**^[AO]^ matrix 264 × 264 (the applied semiempirical method uses 264 AOs to describe the electronic structure of the tetramer).

Further, we contract the **P**^[AO]^ matrix to monomers^73−75^ (denoted with *X*, *Y*) by computing the “fraction of TDM” (FTDM) matrix **F**(22)
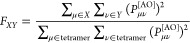
5The diagonal elements *F*_*XX*_ quantify contributions of local excitations (LE) and off-diagonal elements *F*_*XY*_, *Y* ≠ *X* charge-transfer (CT) excitations. For a tetramer, the **F** matrix has a size of 4 × 4. We note that eq 5 does not include the AO overlap matrix, as common in semiempirical theories.

Using the elements of the **F** matrix, we compute the participation number (PN) following the prescription of ref (76) [there the quantity is called “delocalization length” (DL)]
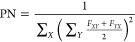
6PN is scalar ranging from 1 (complete exciton localization) to 4 (complete exciton delocalization). We note that the quantity given by eq 6 (or equations of a similar form) is sometimes called “inverse participation ratio” (IPR),^73,77,78^ as was also done in our previous works.^27,47^ However, as recently highlighted by Herbert,^79^ the term IPR may lead to a confusion (see further discussion in the Supplementary Note in the Supporting Information). Therefore, following Alfonso Hernandez *et al.*,^80^ we will call the quantity defined by eq 6 “participation number” (or “PN”) to avoid possible confusions associated with the “IPR”.

We also compute overall measures of LE and CT as
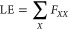
7a
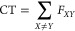
7b

In addition, we introduce the highest (H), intermediate (H–1 and L+1), and lowest (L) monomers through sorting the FTDM diagonal, *F*_H_ > *F*_H–1_ > *F*_L+1_ > *F*_L_, allowing one to judge on exciton localization on single geometry level (for excitons dominated by LE).^27,33,81^ The FTDM and derived quantities (PN, LE, and CT) are calculated along SH trajectories, *i.e.*, they all depend on **R**(*t*). Finally, averaging over a swarm of trajectories (for a given system) is performed.

The monomer dynamics were modeled using rAM1/FOMO–CIS with an active space consisting of HOMO–1 (π), HOMO (n), and LUMO (π*). Three electronic states (S_0_, S_1_, and S_2_) were taken into account, and all 151 trajectories were launched from the S_1_ (nπ*) state.

The rAM1/FOMO–CIS calculations were done with the development version of MOPAC 2002.^82^ The TINKER package was used to handle QM/MM interactions.^83^

## 3 Results and Discussion

### 3.1 Absorption Spectrum and Initial Exciton Localization

**Figure 2:**
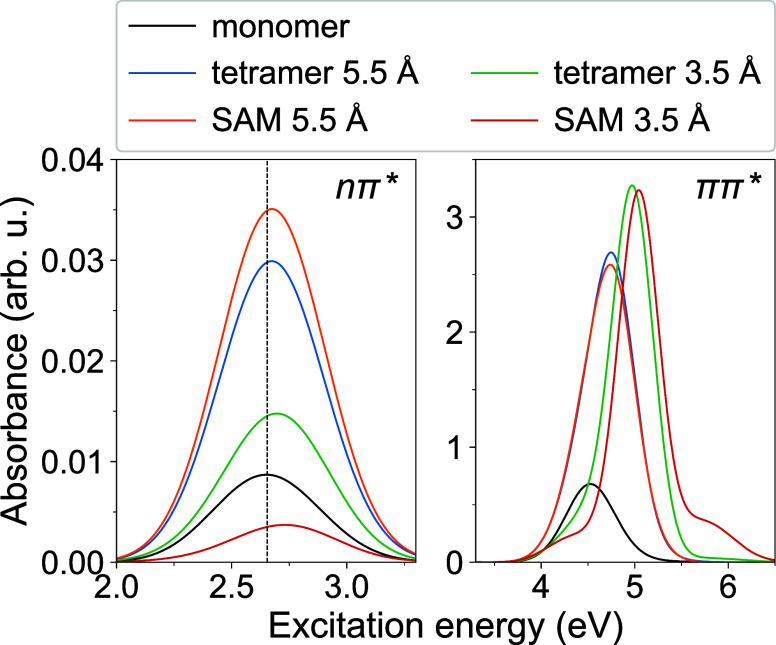
Broadened absorption spectra calculated at the geometries selected from the ground-state Langevin trajectories (151 geometries per system). The nπ* band is shown on the left, and the ππ* band on the right.

The electronic absorption spectra of the models calculated by using eq 1 are shown in Figure 2. These spectra are very similar to those shown in Figure 2 of ref (47) (as expected), but now they are calculated using 151 geometries (per system) instead of 76 used in the previous work.^47^ Excitation energies, oscillator strengths, and orbital contributions to the transitions for model all-*trans* geometries, *i.e.*, in the Franck–Condon region, have already been presented in our previous publication,^47^ see Table S1 in the Supporting Information for ref (47). In the present work, we are concerned with dynamics induced by visible light excitation. Therefore, the nπ* absorption band, located between 2.0 and 3.3 eV (Figure 2, left), is of particular interest here.

As already mentioned above, the nπ* transition of the monomer (the S_0_ → S_1_ transition) is forbidden for the optimized *trans* ground-state geometry processing the *C*_2*h*_ symmetry.^84^ However, the transition acquires some intensity via coupling to vibrations^64^ and is well observed in experiments.^28,29^ Because the transition dipole moment is zero at the equilibrium geometry, it is not possible to predict the changes in excitation energy and oscillator strength upon aggregation using the molecular exciton theory^2^ for the nπ* transition (assuming identical, *C*_2*h*_ geometries of the monomers).

Based on the Langevin, ground-state MD simulations, we observe that the nπ* bands of the tetrameric models are slightly blue-shifted with respect to the monomer, by ∼0.02 eV for models with *a* = 5.5 Å, ∼0.04 eV for tetramer 3.5 Å, and ∼0.08 eV for SAM 3.5 Å. Interestingly, a larger absorbance is observed for models with *a* = 5.5 Å in comparison to those with *a* = 3.5 Å. This effect is opposite to what is seen for the strong ππ* band in the UV region (Figure 2, right). We note that in the simple exciton model (considering an H-aggregate composed of identical monomer geometries with the same nonzero transition dipole moment), a larger enhancement should occur for a smaller separation distance owing to a larger exciton splitting.^25^ As discussed previously for the dimeric models,^57^ the trend in absorbance observed for the nπ* band upon aggregation is determined by the extent of deviation of the monomers forming an aggregate from the equilibrium planar *C*_2*h*_ geometry. To quantify this deviation, we computed the NNCC dihedral angles of the monomers for the studied models for the snapshots selected from the ground-state trajectories (Table S1). The mean NNCC dihedral angles and the corresponding standard deviations decrease in the order SAM 5.5 Å/tetramer 5.5 Å > tetramer 3.5 Å > SAM 3.5 Å, which correlates with the decrease in the nπ* absorbance in this order (Figure 2, left).

**Figure 3:**
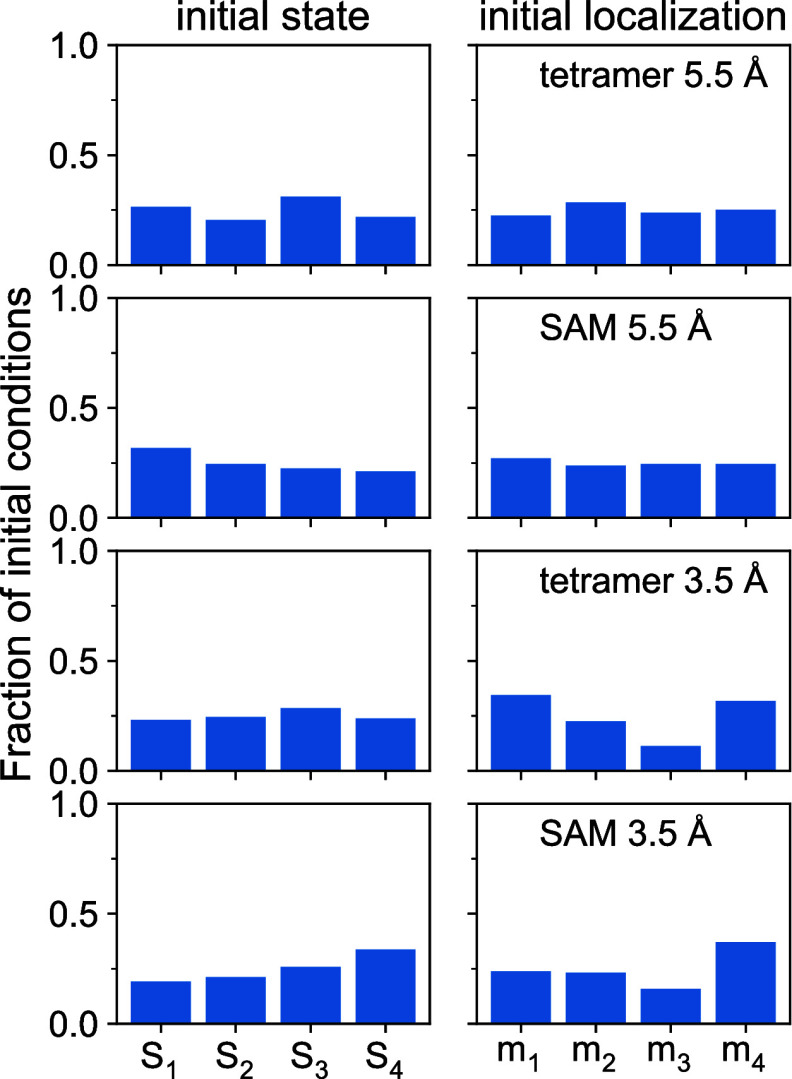
Left column: distributions of initial adiabatic states, which are selected as the brightest states among the nπ* ones. Right column: distributions of initially excited monomers; “m_*X*_” stands for “monomer *X*” with *X* = 1, 2, 3, 4 (see Figure 1 for numbering of the monomers).

Further, the brightest among the nπ* states was selected as an initial electronic state for a SH trajectory. In Figure 3, left, the distributions of the initial adiabatic states are shown. As can be seen, any of the lowest four excited states can be the brightest state depending on an aggregate geometry. The distributions are qualitatively close to the uniform one. Numerically, we find the highest fractions of 0.31 S_3_ for tetramer 5.5 Å, 0.32 S_1_ for SAM 5.5 Å, 0.28 S_3_ for tetramer 3.5 Å, and 0.34 S_4_ for SAM 3.5 Å. These “quasi-uniform” distributions are in contrast to the situation realized for the ππ* excitation, where the S_8_ state is almost always the brightest state.^47^

Furthermore, the nπ* states are strongly localized, *i.e.*, an excitation involves only one monomer, as can be seen in Table 1, where we list ensemble-averaged values of PN and *F*_H_ calculated at the snapshots from the ground-state trajectories, *i.e.*, for the initial conditions for the SH trajectories (*t* = 0). The PN(*t* = 0) values range between ∼1.02 and ∼1.07, and the *F*_H_(*t* = 0) values range between ∼0.97 and ∼0.99. The degree of the initial localization decreases in the order SAM 5.5 Å > tetramer 5.5 Å > tetramer 3.5 Å > SAM 3.5 Å. The strong localization of the brightest nπ* excitons is in agreement with the results of our earlier DFT/TD-DFT study (the TD-DFT PN ≈ 1.1 and *F*_H_ ≈ 0.97 for tetramer 3.5 Å at *T* ≈ 300 K).^27^

**Table 1:** Initial Values of the Participation Number PN(*t* = 0) and the Largest Diagonal Element of FTDM *F*_H_(*t* = 0) Averaged over the Swarm of Initial Geometries (151 per System)

system	PN(*t* = 0)	*F*_H_(*t* = 0)
tetramer 5.5 Å	1.028	0.988
SAM 5.5 Å	1.020	0.991
tetramer 3.5 Å	1.061	0.972
SAM 3.5 Å	1.072	0.967

The initial localization can occur on any of the four monomers of a tetramer, as shown in Figure 3, right, where we plot distributions for initial localization. To do so, the diagonal of the FTDM matrix (**F**) is considered to identify the monomer bearing the highest *F*_*XX*_ value (at a given aggregate geometry). Considering an ensemble of the initial conditions, we count how many times monomer *X* (m_*X*_) traps the exciton. As can be seen in Figure 3, right, the localization distributions are qualitatively more uniform for the models with *a* = 5.5 Å than for the models with *a* = 3.5 Å. Quantitatively, we find the largest fractions of 0.28 m_2_ for tetramer 5.5 Å, 0.27 m_1_ for SAM 5.5 Å, 0.34 m_1_ for tetramer 3.5 Å, and 0.37 m_4_ for SAM 3.5 Å.

### 3.2 Exciton Dynamics

**Figure 4:**
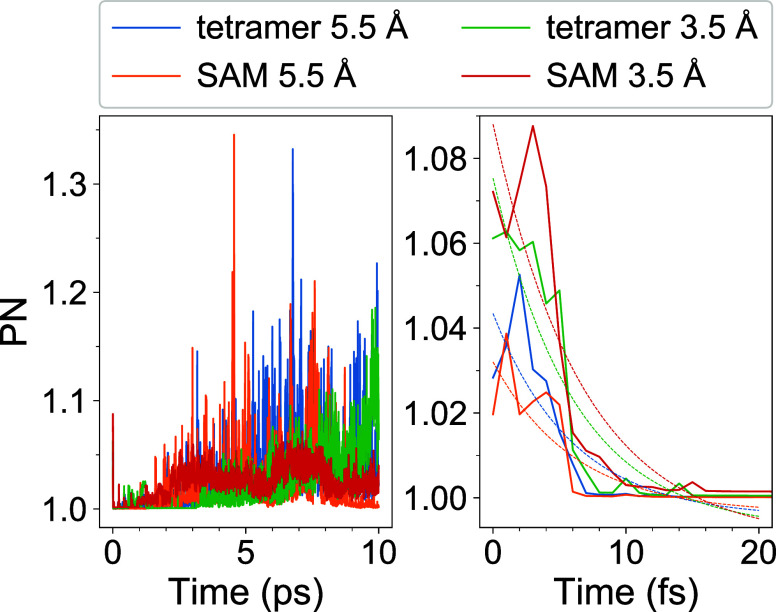
Evolution of PN for the whole simulation period of 10 ps (left) and for the first 20 fs (right) for the studied tetrameric models. The dashed lines in the right panel are the exponential fits defined by eq 8.

The SH MD simulations were performed starting in the brightest nπ* adiabatic state, thus modeling nonadiabatic relaxation after excitation with visible light. The evolution of the ensemble-averaged PN over time is shown in Figure 4. The left panel shows the whole simulation period of 10 ps, whereas the right panel zooms into the first 20 fs. As already described in the previous subsection, the initial excitons are strongly localized with the PN values at *t* = 0 being less than 1.1. Remarkably, during the short-time dynamics (*t* < 10 fs) the excitons become even more localized as can be seen from the right panel of Figure 3, with the PN values approaching ∼1.00. Using a simple exponential fit of the form
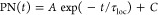
8(applied to the first 20 fs of the simulations) we obtain the exciton localization time constants τ_loc_ presented in the right column of Table 2. These time constants amount to ∼6–7 fs, which means extremely ultrafast further localization (of initially quite strongly localized nπ* excitons) originating from the short-time excited-state dynamics. The fits obtained using eq 8 are also shown in the right panel of Figure 4. We should note that the fits do not reproduce the details of the PN curves obtained from the numerical simulations, but they allow us to obtain a characteristic time of the extremely ultrafast localization process occurring during excited-state dynamics.

**Table 2:** Time Constants for the Studied Systems

system	τ_nπ*_ (fs)	τ_S_0__ (fs)	τ_loc_ (fs)
monomer	1266	1266	
tetramer 5.5 Å	2366	2712	6.0
SAM 5.5 Å	1913	2050	6.1
tetramer 3.5 Å	5890	6235	6.0
SAM 3.5 Å	18,748	20,305	6.8

**Figure 5:**
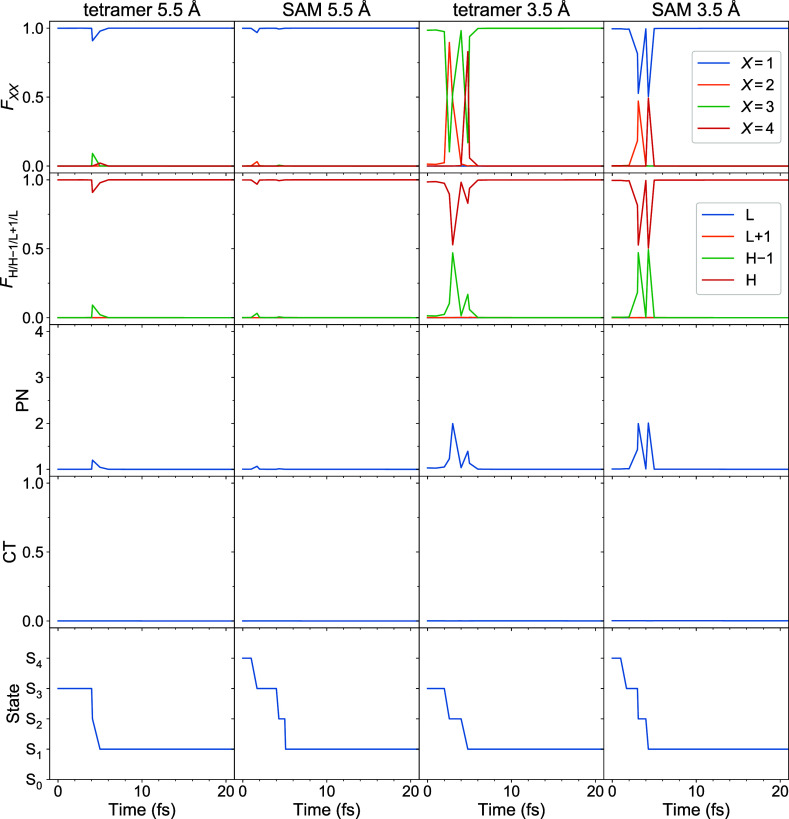
Evolution of *F*_*XX*_ (*X* = 1, 2, 3, 4), *F*_H/H–1/L+1/L_, PN, CT, and the active electronic state for selected single trajectories of all considered tetrameric models.

Further, time evolution of *F*_*XX*_ diagonal matrix elements, highest-to-lowest FTDM values (*F*_H/H–1/L+1/L_), PN, CT, and active adiabatic electronic state for typical single trajectories of the studied systems are shown in Figure 5, for the first 20 fs of dynamics. For the shown trajectories, the initial electronic state is either S_3_ or S_4_ (see the last row of Figure 5). However, internal conversion to the S_1_ state takes only ∼5 fs. The initial exciton is strongly localized on a single fragment—*X* = 1 for all systems except tetramer 3.5 Å, for which *X* = 3 in the given case. The initial localization is transiently perturbed during internal conversion but subsequently recovered, and the exciton stays localized while the aggregate evolves in the S_1_ state. The sudden changes seen in the evolution of **F** matrix elements (and PN) are associated with surface hops occurring along the trajectory (see the last row of Figure 5). We note that no exciton transfer occurs during the short-time dynamics, *i.e.*, the same fragment *X* bears exciton at *t* = 0 and *t* = 20 fs (for the same trajectory). [For few trajectories, the short-time exciton transfer is observed as exemplified in Figure S1.] The transient localization perturbation/delocalization is logically reflected in the PN curves, which deviate from 1 at the corresponding times (see the third row of Figure 5). This behavior correlates with the nonmonotonic short-time evolution of the ensemble-averaged PN curves shown in Figure 4, right. At that, the excitons are composed of local excitations, and CT ≈ 0 (and, hence, LE ≈ 1) throughout the shown time interval, see the fourth row of Figure 5.

**Figure 6:**
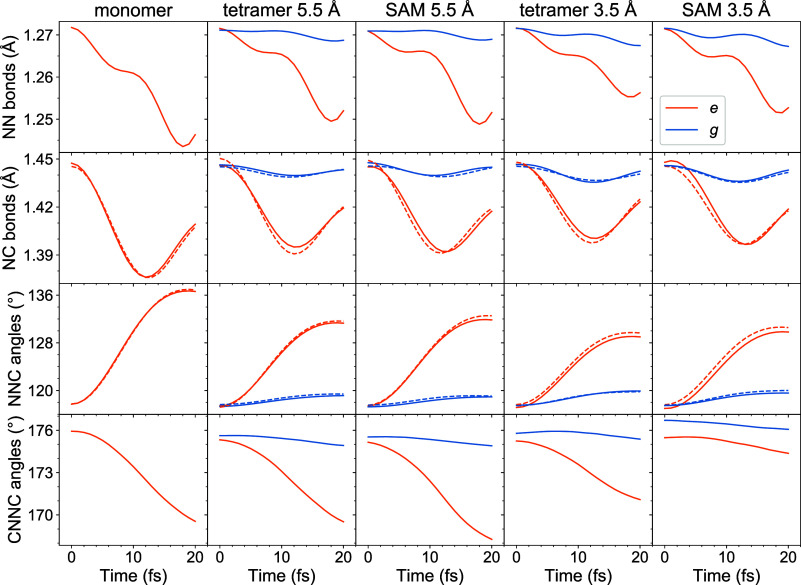
Evolution of selected structural parameters during the short-time dynamics (20 fs) for all considered models. Shown are ensemble-averaged NN and NC bond lengths (the first and the second rows, respectively) as well as NNC and CNNC angles (the third and the fourth rows, respectively). In the case of the NC bonds and NNC angles, the solid lines are for the parameters on the side with the fixed end (“bottom” of the model) and the dashed lines for the parameters on the side with the movable end (“top” of the model). The orange lines are for the excited (*e*) monomers and the blue lines for the nonexcited (*g*) monomers (see the text for further details).

The extremely ultrafast localization is associated with the short-time nuclear dynamics in the nπ* excited states. In Figure 6, we present the evolution of NN and NC bonds as well as NNC angles and CNNC dihedral angles for the first 20 fs of dynamics. The shown curves are averages over the swarm of trajectories. For the tetrameric models, the averaging is performed separately for the excited monomers denoted as *e* (specifically defined as those having the largest *F*_*XX*_ element at *t* = 0) and for nonexcited ones denoted as *g* (with small *F*_*XX*_ values at *t* = 0). For the latter, we additionally average over three *g*-monomers of a given tetramer. In Figure 6, it is seen that NN and NC bonds of the *e*-monomers undergo initial shortening, while NNC angles enlarge and CNNC angles decrease. At that time, changes for the *g*-monomers are much smaller. Thus, the geometry of the excited monomers changes considerably during the short-time dynamics leading to the further exciton localization.

**Figure 7:**
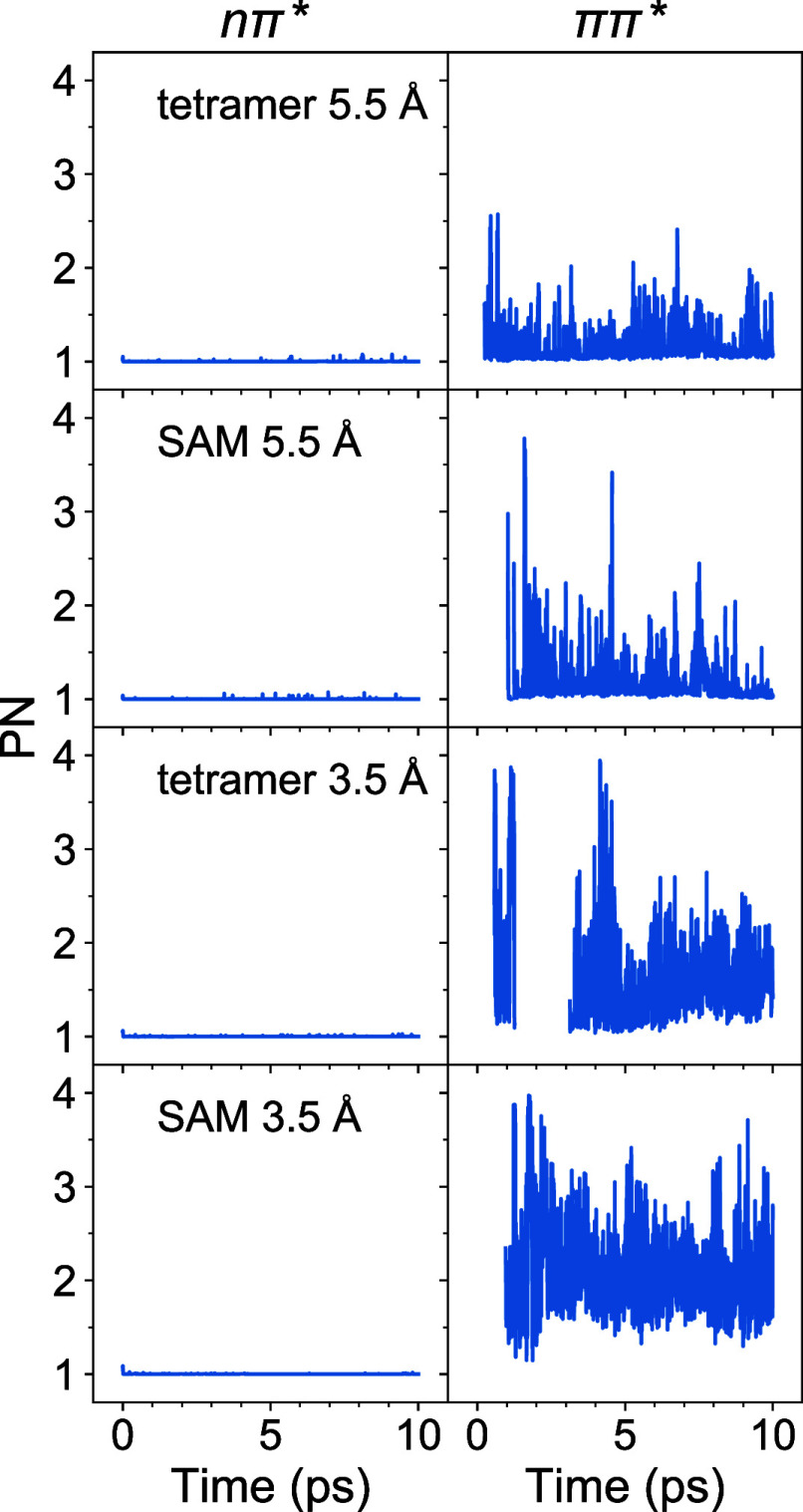
PN for nπ* (left) and ππ* (right) excitons.

**Figure 8:**
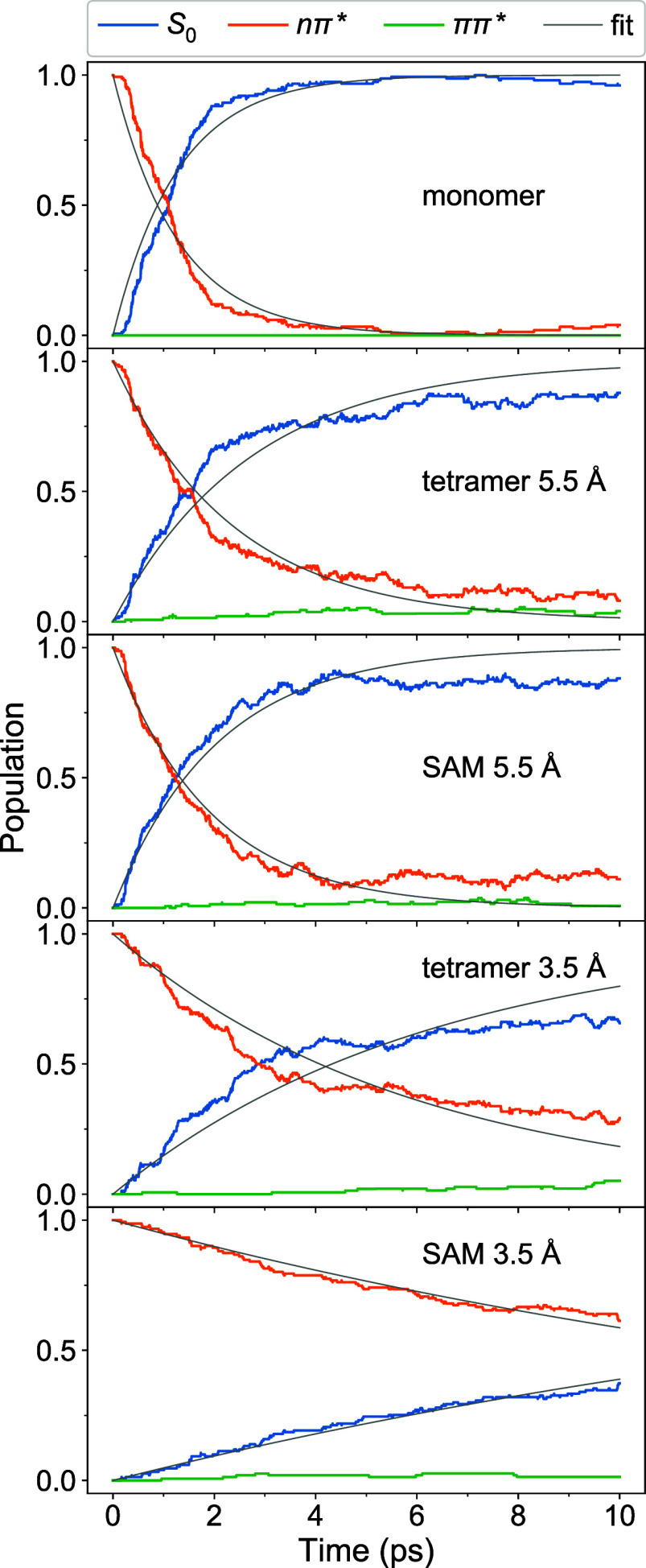
Populations of the S_0_, nπ*, and ππ* states for the studied systems. The gray lines show exponential fits defined in eq 9a, 9b.

Further, at longer times, the PN curves become more “noisy” and reach larger values (Figure 4, left), with the following maxima: PN_max_ ≈ 1.33 (6767 fs) for tetramer 5.5 Å, PN_max_ ≈ 1.35 (4560 fs) for SAM 5.5 Å, PN_max_ ≈ 1.19 (9875 fs) for tetramer 3.5 Å, and PN_max_ ≈ 1.07 (7108 fs) for SAM 3.5 Å. To understand this behavior, we computed the PN curves separately for the nπ* and ππ* manifolds, *i.e.*, averaging (at time *t*) only over trajectories residing in the corresponding manifold at time *t*. We note that the ππ* states located above the nπ* states are slightly populated in the course of the nonadiabatic dynamics (see Figure 8 in the next subsection). The nπ* and ππ* PN curves are shown in Figure 7. It is seen that the nπ* curves are close to 1 at all times (Figure 7, left column). Quantitatively, the nπ* PN values are <1.09 ∀*t* for all the models, and the temporal mean (over time of 10 ps) nπ* PN values are 1.0007, 1.0004, 1.0010, and 1.0018 for tetramer 5.5 Å, SAM 5.5 Å, tetramer 3.5 Å, and SAM 3.5 Å, respectively. The ππ* PN curves, on the other hand, extend up to PN ≈ 4 (Figure 7, right column), with the temporal mean values of 1.14, 1.15, 1.60, and 2.08 for tetramer 5.5 Å, SAM 5.5 Å, tetramer 3.5 Å, and SAM 3.5 Å, respectively. Thus, the longer time behavior of the overall PN curves (Figure 4, left) arises from the interplay of the strongly localized nπ* states and more delocalized ππ* states.

Next, considering the entire simulation period of 10 ps, we find virtually no exciton transfer (at longer times) while the systems evolve in the nπ* manifold. There are only three trajectories demonstrating the opposite (see Figure S2). The very rare occurrence of the nπ* exciton transfer is in agreement with our previous simulations of the ππ* dynamics (where the nπ* states are reached via internal conversion from the ππ* states).^47^ Moreover, it agrees with the previous simulations of Sangiogo Gil *et al.* based on an exciton model.^46^ However, for some trajectories the exciton transfer occurs via nπ* → ππ* → nπ* or nπ* → S_0_ → nπ* pathways (see Figure S3). These pathways involve upward hops that require a certain amount of kinetic energy to occur since the change in the potential energy caused by the hop should be compensated to conserve the total energy. In this respect, we note that there is no energy dissipation to the environment in our models; therefore, the upward hops are facilitated in the present simulations.

Lastly, there is virtually no participation of charge-transfer excitations during the dynamics initiated in the nπ* states, as already discussed for the short-time dynamics above. In fact, we find CT ≈ 0 and LE ≈ 1 throughout the whole simulation period of 10 ps for all systems (Figure S4). For *a* = 3.5 Å, especially for SAM 3.5 Å, there is a clear difference to the dynamics initiated in the ππ* manifold, for which CT plays a bigger role (cf. Figure 5 of ref (47)). For *a* = 5.5 Å, the situation is similar for both nπ*- and ππ*-initiated dynamics, with CT ≈ 0 and LE ≈ 1 throughout the entire simulation period (cf. Figure S6 of ref (47)).

### 3.3 Excited-State Lifetimes and Quantum Yields of *trans*-to-*cis* Isomerization

The electronic state populations are listed in Figure 8. The nπ* manifold comprises states S_1_–S_4_ and ππ* manifold S_5_–S_8_ of the tetrameric models. For the monomer, nπ* includes S_1_ and ππ* S_2_.

The nπ* and S_0_ population curves were fitted using a simple monoexponential decay model
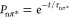
9a
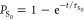
9b

The fitted curves are shown as thin gray lines in Figure 8, and the corresponding time constants τ_nπ*_ and τ_S_0__ are listed in Table 2. For the monomer, we find the nπ* lifetime τ_nπ*_ of ∼1.3 ps. This lifetime is longer than that calculated by Cantatore *et al.*, ∼0.4 ps, using rAM1/FOMO–CI with more active orbitals and multiple excitations.^85^ Gámez *et al.* have obtained a similar lifetime of ∼0.3 ps from OM2/MRCI SH simulations.^86^ Yu *et al.* have calculated a lifetime of ∼0.8 ps using global switching SH and state-averaged CASSCF.^87^ In contrast, a much longer lifetime of ∼6 ps was reported by Yu *et al.* based on FOMO-*hh*-TDA AIMS simulations.^88^ [We note though that the result seems to be very sensitive to the electronic temperature,^89^ as the same authors previously found a lifetime of ∼0.2 ps using nominally the same method^90^.] In any case, we will use the ∼1.3 ps to compare with because the calculations for the tetrameric models are performed using CIS on supermolecular orbitals originating from the three orbitals of the monomer (HOMO–1, HOMO, and LUMO). We also note that the time constant for the ground-state recovery, τ_S_0__, is the same as the nπ* excited-state lifetime (Table 2) because the ππ* state of the monomer is not populated during the dynamics (at the present level of theory), see Figure 8, top.

For the tetrameric models, the nπ* lifetimes are prolonged, moderately for models with *a* = 5.5 Å (∼2.4 ps for tetramer 5.5 and ∼1.9 ps for SAM 5.5 Å), stronger for tetramer 3.5 Å (∼5.9 ps), and the most for SAM 3.5 Å (∼18.7 ps). The long nπ* lifetime for tightly packed SAM 3.5 Å is in qualitative agreement with previous simulations of azobenzene-containing SAMs.^46,91^ At that, the internal conversion to the S_1_ state is extremely fast for all studied models—S_1_ is populated almost completely within ≲10 fs (see Figure S5). This result may be expected because the nπ* exciton splittings are small, and it is again in agreement with ref (46), where the population transfer to the S_1_ state for a model of 12 QM molecules occurs within 40 fs.

Further, for the tetrameric models, we observe that the ππ* states are slightly populated during dynamics. As a result of strong exciton splitting for the ππ* states, the lowest ππ* state is closer to the nπ* states than in the monomer case, thus leading to a smaller energy gap between the nπ* and ππ* manifolds. Moreover, the tetrameric models possess a larger initial kinetic energy, which enables upward surface hops in contrast to the monomer, for which these hops are frustrated. The population of the ππ* states is responsible for ground-state return time constants (τ_S_0__) being longer than the nπ* excited state lifetimes (τ_nπ*_) (for a given tetrameric system), see Table 2. Namely, we find τ_S_0__ of ∼2.7–2.1, ∼6.2, and ∼20.3 ps for tetramer 5.5 Å, SAM 5.5 Å, tetramer 3.5 Å, and SAM 3.5 Å, respectively. Moreover, the population of the ππ* states may lead to the nπ* → ππ* → nπ* exciton-transfer pathway as discussed above (see Figure S3).

**Figure 9:**
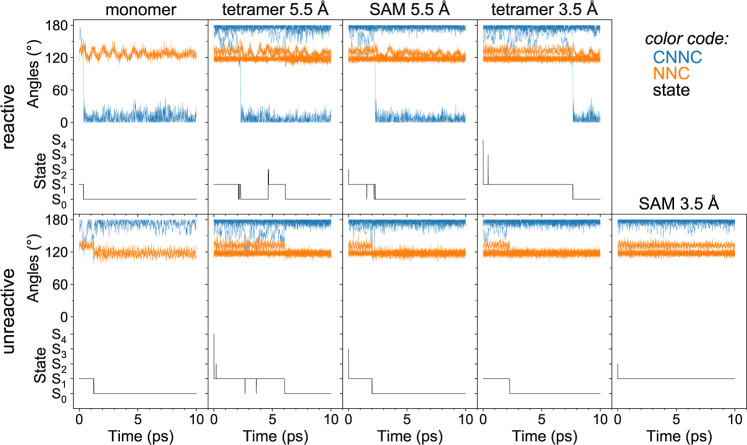
Evolution of CNNC dihedral angles and NNC angles for selected reactive (top row) and unreactive (bottom row) trajectories, for all studied models. For each tetrameric model, four CNNC angles (all in blue) and eight NNC angles (all in orange) are plotted. [For the monomer, one CNNC (blue) and two NNC (orange) angles are shown.] The current state as a function of time is shown at the bottom of each subplot.

Lastly, we computed the quantum yields of the *trans* → *cis* isomerization. To do so, the reactive trajectories were identified based on the evolution of the CNNC dihedral angles. The examples of the reactive (*trans* → *cis*) and unreactive (*trans* → *trans*) trajectories are shown in Figure 9. The isomerization event (observed for the reactive trajectories) corresponds to the large change of the CNNC dihedral from values close to ∼180° (*trans* isomer) to values close to ∼0° (cis isomer), whereas for the unreactive trajectories the CNNC angles stay large (close to ∼180°) throughout the internal conversion process. At that, initial excitation always leads to a decrease of the CNNC angle and an increase of NNC angles for the excited molecule/monomer. For the reactive trajectories, the drop in the CNNC curve (corresponding to the switching) occurs simultaneously with transition to the ground state (S_0_). Moreover, for the shown trajectories, we see that this happens at ∼400 fs for the monomer, at ∼2.3 ps for the models with *a* = 5.5 Å, and at ∼7.7 ps for tetramer 3.5 Å (no reactive trajectories were found for SAM 3.5 Å). This reflects the slower kinetics of tetrameric models (especially for *a* = 3.5 Å), as observed in Figure 8 [see also the unreactive trajectory for SAM 3.5 (Figure 9, bottom right), which remains in the S_1_ state until the end of the simulation (10 ps)].

In this work, we simulated 151 trajectories for each of the studied systems, whereas only 76 trajectories per system were simulated in our previous work on the ππ*-initiated dynamics (except for tetramer 5.5 Å, for which additional 75 trajectories were run in ref (47) to check the effect of enlargement of the ensemble on the quantum yield). In Table 3, we report nπ* (this work) and ππ* (ref (47)) quantum yields for batches of 76 trajectories and 151 trajectories (for the latter, in the case of the ππ* excitation, only for the monomer and tetramer 5.5 Å). [In this work, we performed additional calculations for the monomer to calculate the ππ* quantum yield based on 151 trajectories.]

**Table 3:** Quantum Yields Φ ± ΔΦ (in %) of the *trans* → *cis* Isomerization after nπ* and ππ* Excitations for the Studied Systems, for the 151 Trajectory Batch (Left) and the 76 Trajectory Batch (Right)^a^

	151 trajectories		76 trajectories	
system	nπ*	ππ*	nπ*	ππ*
monomer	13.9 ± 2.8 (151)	10.7 ± 2.5 (149)	18.4 ± 4.4 (76)	14.7 ± 4.1 (75)^b^
tetramer 5.5 Å	8.3 ± 2.3 (144)	4.1 ± 1.8 (123)^b^	5.6 ± 2.7 (71)	5.1 ± 2.9 (59)^b^
SAM 5.5 Å	7.1 ± 2.2 (141)		6.9 ± 3.0 (72)	4.9 ± 2.8 (61)^b^
tetramer 3.5 Å	8.1 ± 2.4 (135)		7.1 ± 3.1 (70)	4.6 ± 2.6 (65)^b^
SAM 3.5 Å	0 (80)		0 (41)	0 (66)^b^

a The number of trajectories which reached the ground state (*N*_t_) is shown in parentheses.

b From ref (47).

First of all, comparing within a given batch (76 or 151 trajectories), we see that the nπ* quantum yield of the monomer is higher than the ππ* quantum yield, namely, ∼ 14 vs ∼11% (151 trajectories) and ∼18 vs ∼15% (76 trajectories). While this is correct qualitatively, we see that the nπ* quantum yield is only 1.2–1.3 times larger than the ππ* quantum yield instead of a known factor of ∼2.^88,92^ In this respect, we note that in this work, we use FOMO–CIS, *i.e.*, only single excitations within an active space of HOMO–1, HOMO, and LUMO are taken into account. To reveal the role of double excitations, we performed SH simulations with FOMO–CISD within the same active space, resulting in nine Slater determinants used to construct wave functions. These calculations resulted in a quantum yield (for the monomer) of ∼30% (29.8 ± 3.7% for 151 trajectories and 30.3 ± 5.3% for 76 trajectories). The corresponding nπ* lifetime is 1092 fs. Thus, while inclusion of double excitations considerably increases the quantum yield [from ∼14% (CIS) to ∼30% (CISD) in the case of the full set of 151 trajectories], the nπ* lifetime still remains long, ∼1 ps [it decreases from 1266 fs (CIS) to 1092 fs (CISD), see Figure S6 for S_1_ populations calculated with both methods]. Expansion of the active space is therefore needed to obtain a shorter lifetime compatible with earlier works.^85,86,90^

Further, similarly to the case of the ππ* excitation,^47^ we observe smaller quantum yields when going from the monomer to the tetrameric models (Table 3). Specifically, the nπ* quantum yields are ∼7–8% for models with *a* = 5.5 Å and tetramer 3.5 Å and 0 for SAM 3.5 Å. The reduction factor “monomer/tetramer” is ∼2 (considering the batch of 151 trajectories). Compared to the ππ* quantum yields, the nπ* quantum yields are slightly higher, as is the case for the monomer (Table 3) (except for SAM 3.5 Å, for which Φ = 0 in either case). Here, we should note that previously, for a dimeric SAM model with *a* = 4.0 Å, the opposite was found, *i.e.*, Φ_nπ*_ ≈ 3% and Φ_ππ*_ ≈ 9%.^58^ There can be several reasons responsible for this discrepancy, including different electronic structure methods (in terms of the size of the active space and allowed excitations), the use or disuse of the added potential,^61^ and the size of the QM part. The understanding of this problem would require further simulations going beyond the scope of the present work.

Next, for the reactive trajectories, it is interesting to analyze which monomer undergoes isomerization. We note that, for the reactive trajectories, usually only one of the four monomers switches to the cis form (only three trajectories exhibit additional isomerizations caused by repopulation of excited states from the ground state). The isomerization of only one monomer may actually be expected because the initial exciton (at *t* = 0) is quite localized on a single monomer, which then relaxes to the ground state either experiencing *trans* → *cis* switching or returning back to the *trans* form. For tetramer 3.5 Å, out of 11 reactive trajectories, we find that 4, 1, 0, and 6 correspond to isomerization of monomers 1, 2, 3, and 4, respectively (see Figure 1 for numbering of the monomers). Thus, for tetramer 3.5 Å, there is a preference for isomerization of end molecules (1 and 4). For tetramer 5.5 Å, out of 12 reactive trajectories, 3, 3, 3, and 3 show isomerization of monomers 1, 2, 3, and 4, respectively. In this case, we find a uniform “distribution”. However, the overall quantum yields of tetramers 3.5 and 5.5 Å are very similar (both ∼8%). Thus, despite the fact that every QM molecule can isomerize in tetramer 5.5 Å, it turns out that azobenzene isomerization ability is affected at *a* = 5.5 Å. For SAM 5.5 Å, out of 10 reactive trajectories, we find 4, 3, 2, and 1 trajectories with switching of monomers 1, 2, 3, and 4, respectively. In this case, the “distribution” is asymmetric which can originate from too few members of the sample.

Finally, enlargement of the batch size from 76 to 151 trajectories leads to the following changes in the quantum yields: −4.5% for monomer upon nπ* excitation and −4.0% for monomer upon ππ* excitation; +2.7% for tetramer 5.5 Å (nπ*) and −1.0% for tetramer 5.5 Å (ππ*); +0.2% for SAM 5.5 Å (nπ*); and +1% for tetramer 3.5 Å (nπ*). For SAM 3.5 Å, no isomerization is observed, irrespective of excitation (nπ* or ππ*) and the batch size.

## 4 Conclusions

We studied the nonadiabatic dynamics of tetrameric aggregates of azobenzene upon nπ* excitation using the rAM1/FOMO–CIS electronic structure approach combined with the SH scheme for modeling nonadiabatic dynamics. The exciton dynamics of the nπ* excitons excited with visible light are the focus of the present study. These dynamics were revealed using the TDM analysis.

We found that the initial excitons (computed at the initial geometries from ground-state Langevin MD) are strongly localized. However, they undergo even further extremely ultrafast localization during the first femtoseconds of nonadiabatic dynamics with a time constant of 6–7 fs. After that, the nπ* excitons stay localized throughout the dynamics and normally no exciton transfer occurs between monomers while aggregates evolve in the nπ* manifold. Only a few trajectories demonstrated exciton transfer during dynamics in the nπ* states. In addition, we observed that the exciton transfer may be realized through population of the higher lying ππ* states or through upward hops from the ground state (reached in turn via internal conversion) to excited states.

The nπ* *trans* → *cis* isomerization quantum yields are lower by a factor of about two for free/not strongly constrained tetramers than for the monomer, and no switching is observed for the most tightly packed model (SAM 3.5 Å). The nπ* quantum yields for the aggregates are found to be slightly larger than the ππ* quantum yields (obtained at the same level of theory in ref (47)).

## Supporting Information Available

Supplementary Note about IPR, mean NNCC and CNNC dihedral angles for initial geometries, examples of trajectories demonstrating exciton transfer, LE and CT curves, populations of the individual states, and S_1_ populations for the monomer calculated with FOMO–CIS and FOMO–CISD (PDF)
