# Supplementary material for: Visible Light Induced Exciton Dynamics and trans-to-cis Isomerization in Azobenzene Aggregates: Insights from Surface Hopping/Semiempirical Configuration Interaction Molecular Dynamics Simulations
Source: ACS Omega. 2024 Feb 9;9(7):8520–32. doi: 10.1021/acsomega.3c09900 (PMC10882624; doi:10.1021/acsomega.3c09900)
Supplement: Supplementary file 1 — ao3c09900_si_001.pdf [file ao3c09900_si_001.pdf]

Supporting Information for “Visible-Light-Induced Exciton Dynamics and Trans-to-Cis Isomerization in Azobenzene Aggregates: Insights from Surface Hopping / Semiempirical Configuration Interaction Molecular Dynamics Simulations”

Evgenii Titov*

University of Potsdam, Institute of Chemistry, Theoretical Chemistry, Karl-Liebknecht-Straße 24-25, 14476 Potsdam, Germany

E-mail: titov@uni-potsdam.de

Supplementary Note

Indeed, following ref. S1, complete localization corresponds to a participation ratio (PR) of $1/n$, and IPR $\equiv 1/\mathrm{PR} = n$ (with n being the number of monomers), whereas for complete delocalization PR $= 1$ (and IPR $= 1$ as well). On the other hand, according to Scholes,[S2] localization corresponds to IPR $= 1$ and delocalization to IPR $= 1/n$, and the delocalization length is given by DL $= 1/\mathrm{IPR}$. Moreover, comparing the definition of PR given by Bell[S3] to the definition of IPR by Thouless,[S4] one can see that IPR $\neq 1/\mathrm{PR}$, instead $\mathrm{PR} \cdot \mathrm{IPR} = 1/n$ (see also ref. S5). It should be noted though that Thouless[S4] referred to the definition given by Bell and Dean in ref. S6 that differs from that in ref. S3 by a factor of $1/n$, thus leading to $\mathrm{PR} \cdot \mathrm{IPR} = 1$ (see also eqs. (3) and (4) in ref. S7).

Table S1: Mean values of NNCC and CNNC dihedral angles with standard deviations (in parentheses) in degrees for initial geometries (selected from the ground-state Langevin MD trajectories). $\mathrm{NNCC}_i^{(j)}$ ($i = 1, 2, 3, 4$; $j = 1, 2$) is the NNCC dihedral angle of monomer i on side j; $j = 1$ corresponds to the side with a movable end ("top" of the aggregate, cf. Fig. 1 of the main text) and $j = 2$ to the side with a fixed end ("bottom" of the aggregate). For each side, the smaller of two NNCC angles (defined at the geometry of the first snapshot) is chosen, *i.e.*, the one which is closer to 0° than to 180°. CNNC_i is the CNNC dihedral angle of monomer i. All dihedrals are defined to lie in the [0°,180°] interval.

angle	monomer	tetramer 5.5 Å	SAM 5.5 Å	tetramer 3.5 Å	SAM 3.5 Å
$\mathrm{NNCC}_1^{(1)}$	18 (15)	18 (14)	22 (17)	16 (12)	12 (9)
$\mathrm{NNCC}_2^{(1)}$		20 (15)	19 (13)	14 (11)	13 (8)
$\mathrm{NNCC}_3^{(1)}$		19 (13)	19 (14)	13 (10)	13 (9)
$\mathrm{NNCC}_4^{(1)}$		16 (12)	21 (17)	16 (11)	12 (9)
$\mathrm{NNCC}_1^{(2)}$	43 (55)[a]	18 (14)	20 (14)	16 (12)	13 (9)
$\mathrm{NNCC}_2^{(2)}$		19 (15)	19 (14)	16 (11)	11 (8)
$\mathrm{NNCC}_3^{(2)}$		19 (15)	19 (14)	15 (11)	13 (9)
$\mathrm{NNCC}_4^{(2)}$		20 (14)	21 (15)	15 (12)	11 (9)
CNNC_1	176 (3)	176 (3)	176 (3)	176 (3)	176 (3)
CNNC_2		176 (3)	176 (3)	176 (3)	176 (2)
CNNC_3		176 (3)	176 (3)	176 (3)	177 (2)
CNNC_4		175 (3)	175 (4)	175 (4)	176 (3)

[a] This large value originates from rotation of the phenyl ring around the CN bond during Langevin dynamics.

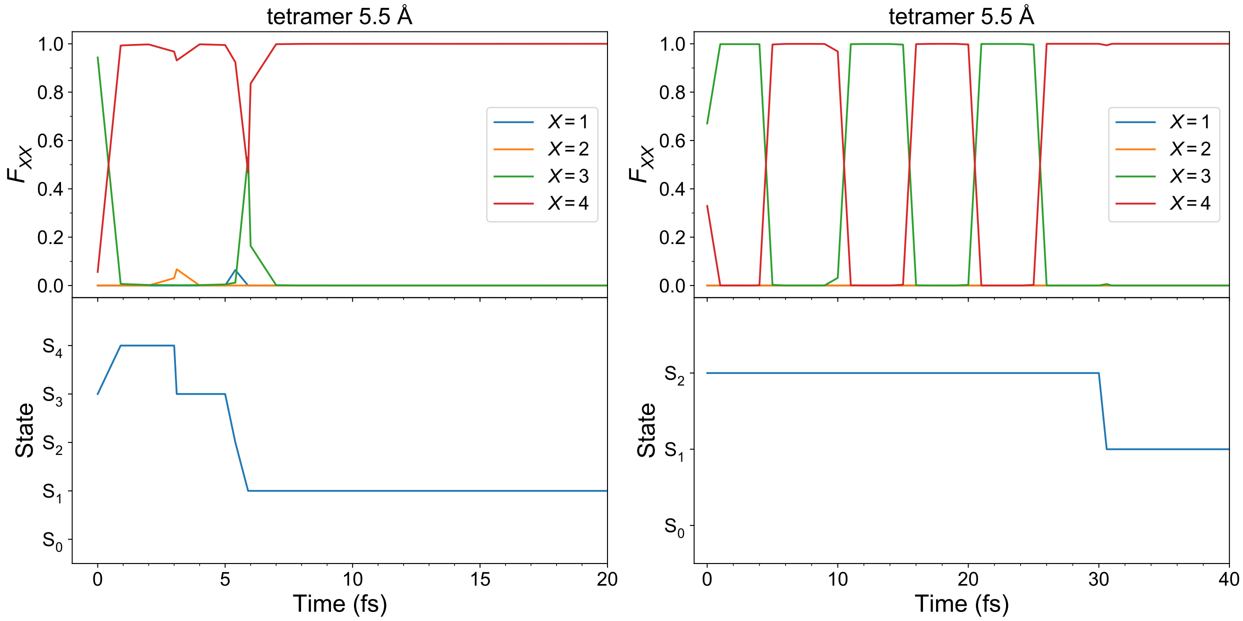

Figure S1: Examples of trajectories showing exciton transfer during short-time dynamics in the $n\pi^*$ manifold. Shown are F_{XX} and active state.

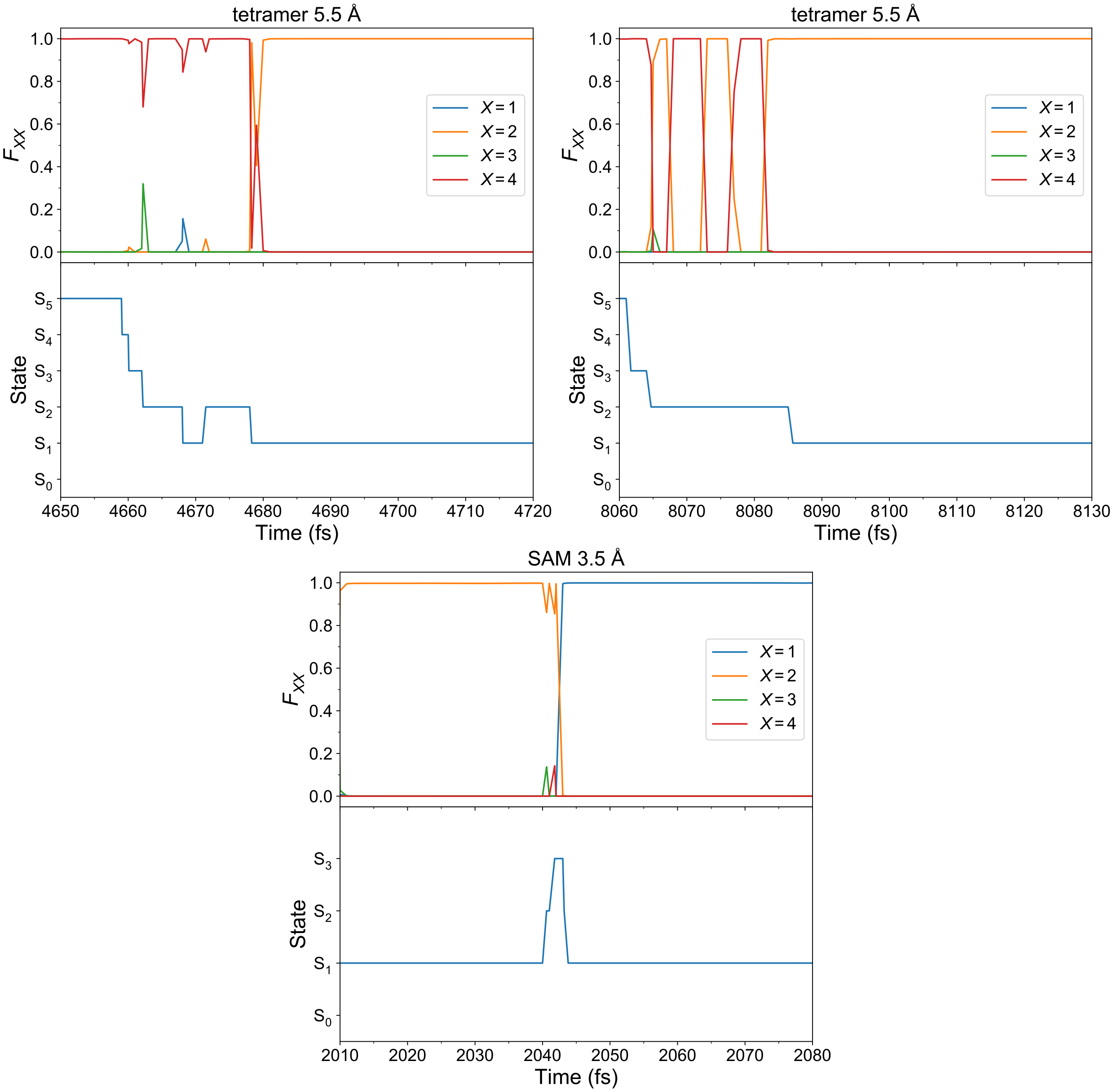

Figure S2: Trajectories demonstrating exciton transfer in the $n\pi^*$ manifold at longer times. Shown are F_{XX} and active state.

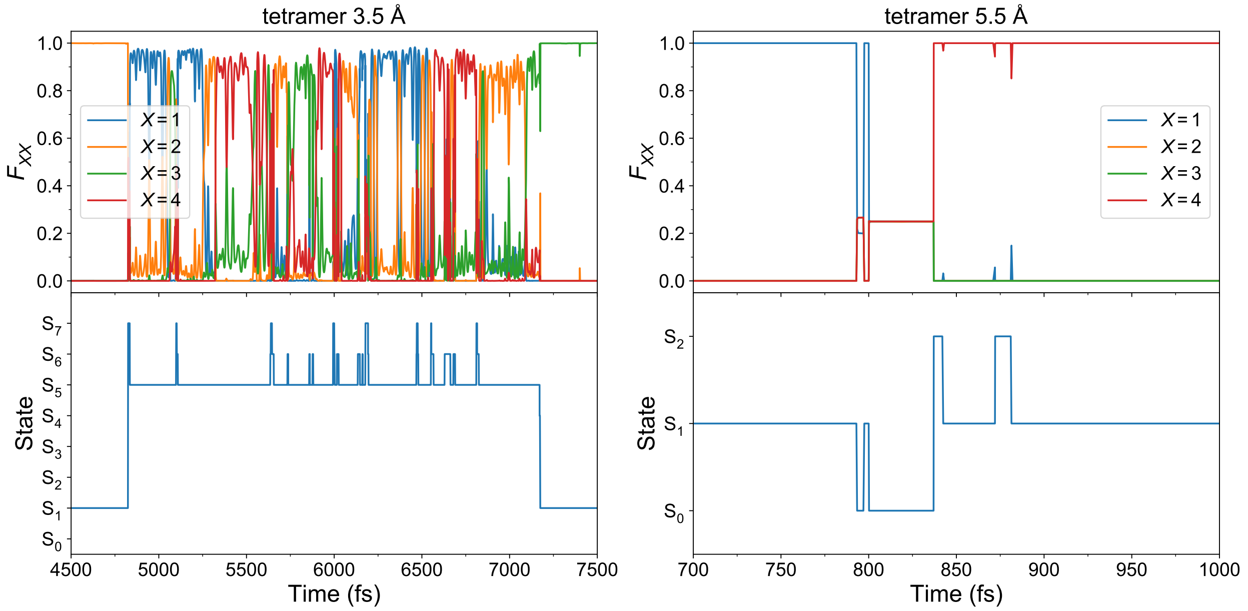

Figure S3: Examples of trajectories showing exciton transfer via the $n\pi^* \rightarrow \pi\pi^* \rightarrow n\pi^*$ pathway (left) and $n\pi^* \rightarrow S_0 \rightarrow n\pi^*$ pathway (right). Shown are F_{XX} and active state.

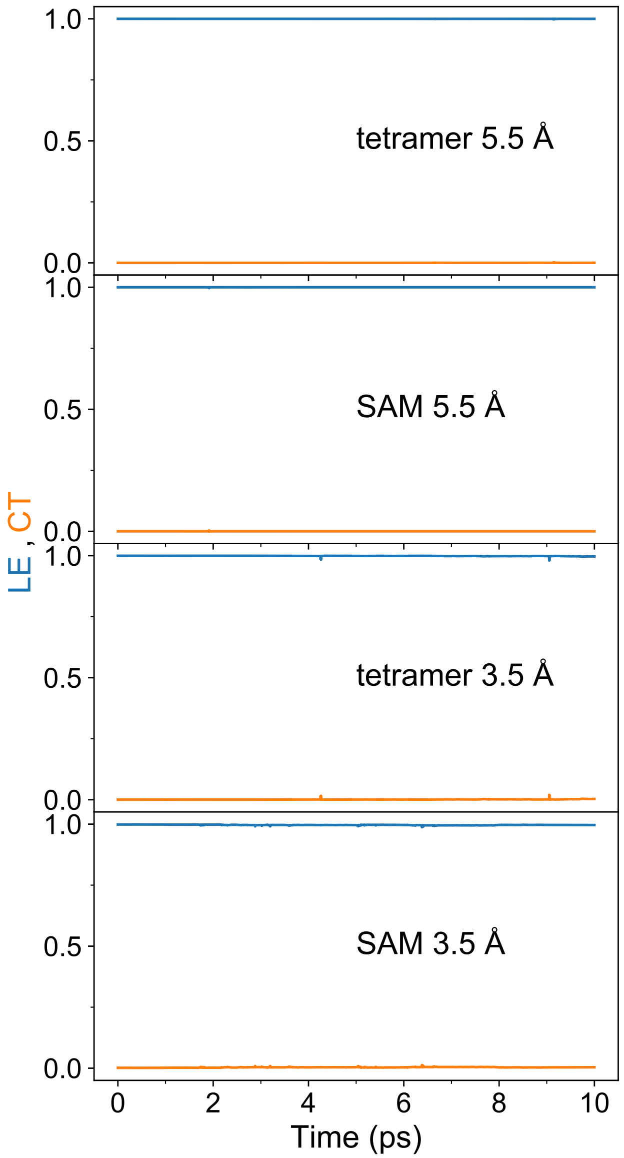

Figure S4: Evolution of LE and CT for the studied models.

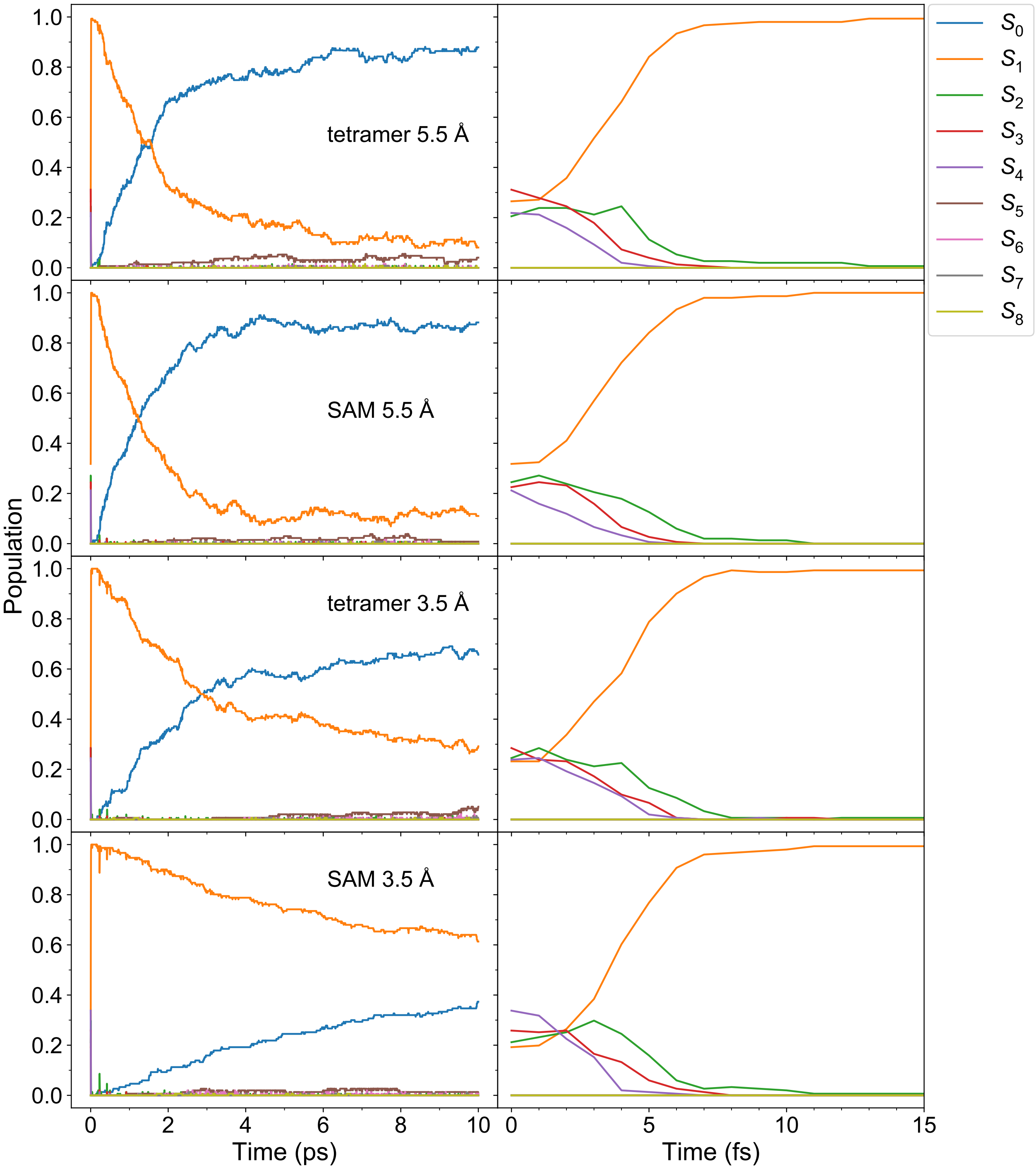

Figure S5: Populations of the individual states shown for the total simulation period of 10 ps (left column) and for the first 15 fs (right column).

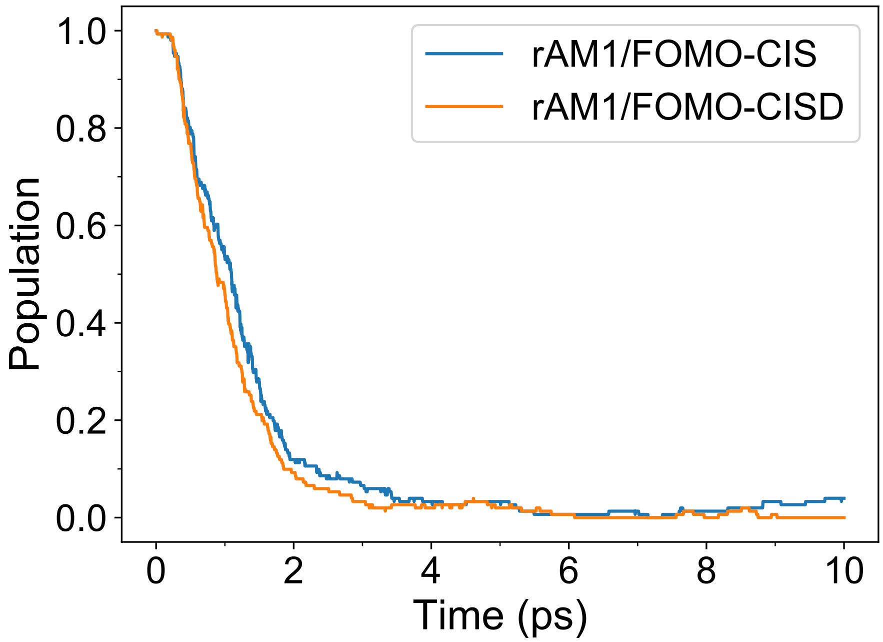

Figure S6: S_1 populations for the monomer calculated with rAM1/FOMO-CIS and rAM1/FOMO-CISD using the active space consisting of HOMO−1, HOMO, and LUMO.

References

(S1) Clark, T. B. P.; Maestro, A. D. Moments of the inverse participation ratio for the Laplacian on finite regular graphs. *J. Phys. A: Math. Theor.* **2018**, *51*, 495003.

(S2) Scholes, G. D. Limits of exciton delocalization in molecular aggregates. *Faraday Discuss.* **2020**, *221*, 265–280.

(S3) Bell, R. J. The dynamics of disordered lattices. *Rep. Prog. Phys.* **1972**, *35*, 1315.

(S4) Thouless, D. Electrons in disordered systems and the theory of localization. *Phys. Rep.* **1974**, *13*, 93–142.

(S5) Wegner, F. Inverse participation ratio in 2+ ε dimensions. *Z. Phys. B: Condens. Matter* **1980**, *36*, 209–214.

(S6) Bell, R. J.; Dean, P. Atomic vibrations in vitreous silica. *Discuss. Faraday Soc.* **1970**, *50*, 55–61.

(S7) Bell, R. J.; Dean, P.; Hibbins-Butler, D. C. Localization of normal modes in vitreous silica, germania and beryllium fluoride. *J. Phys. C: Solid State Phys.* **1970**, *3*, 2111.
